# Prospecting for rare earth element (hyper)accumulators in the Paris Herbarium using X-ray fluorescence spectroscopy reveals new distributional and taxon discoveries

**DOI:** 10.1093/aob/mcae011

**Published:** 2024-02-03

**Authors:** Léo Goudard, Damien Blaudez, Catherine Sirguey, Imam Purwadi, Vanessa Invernon, Germinal Rouhan, Antony van der Ent

**Affiliations:** Université de Lorraine, INRAE, LSE, F-54000, Nancy, France; Université de Lorraine, CNRS, LIEC, F-54000, Nancy, France; Université de Lorraine, INRAE, LSE, F-54000, Nancy, France; Centre for Mined Land Rehabilitation, Sustainable Minerals Institute, The University of Queensland, Brisbane, Australia; Institut de Systématique, Evolution, Biodiversité (ISYEB), Muséum national d’Histoire naturelle, CNRS, Sorbonne Université, École Pratique des Hautes Études, Université des Antilles, Paris, France; Institut de Systématique, Evolution, Biodiversité (ISYEB), Muséum national d’Histoire naturelle, CNRS, Sorbonne Université, École Pratique des Hautes Études, Université des Antilles, Paris, France; Université de Lorraine, INRAE, LSE, F-54000, Nancy, France; Laboratory of Genetics, Wageningen University and Research, Wageningen, The Netherlands

**Keywords:** Hyperaccumulators, phylogenetic diversity, XRF technology

## Abstract

**Background:**

Rare earth elements (REEs) are increasingly crucial for modern technologies. Plants could be used as a biogeochemical pathfinder and a tool to extract REEs from deposits. However, a paucity of information on suitable plants for these tasks exists.

**Methods:**

We aimed to discover new REE-(hyper)accumulating plant species by performing an X-ray fluorescence (XRF) survey at the Herbarium of the Muséum national d’Histoire naturelle (MNHN, Paris, France). We selected specific families based on the likelihood of containing REE-hyperaccumulating species, using known taxa that accumulate REEs. A total of 4425 specimens, taken in the two main evolutionary lineages of extant vascular plants, were analysed, including the two fern families Blechnaceae (*n* = 561) and Gleicheniaceae (*n* = 1310), and the two flowering plant families Phytolaccaceae (*n* = 1137) and Juglandaceae (*n* = 1417).

**Key Results:**

Yttrium (Y) was used as a proxy for REEs for methodological reasons, and a total of 268 specimens belonging to the genera *Blechnopsis* (*n* = 149), *Dicranopteris* (*n* = 75), *Gleichenella* (*n* = 32), *Phytolacca* (*n* = 6), *Carya* (*n* = 4), *Juglans* (*n* = 1) and *Sticherus* (*n* = 1) were identified with Y concentrations ranging from the limit of detection (LOD) >49 µg g^−1^ up to 1424 µg g^−1^. Subsequently, analysis of fragments of selected specimens by inductively coupled plasma atomic emission spectroscopy (ICP-AES) revealed that this translated to up to 6423 µg total REEs g^−1^ in *Dicranopteris linearis* and up to 4278 µg total REEs g^−1^ in *Blechnopsis orientalis* which are among the highest values ever recorded for REE hyperaccumulation in plants. It also proved the validity of Y as an indicator for REEs in XRF analysis of herbarium specimens. The presence of manganese (Mn) and zinc (Zn) was also studied by XRF in the selected specimens. Mn was detected in 1440 specimens ranging from the detection limit at 116 µg g^−1^ up to 3807 µg g^−1^ whilst Zn was detected in 345 specimens ranging from the detection limit at 77 µg g^−1^ up to 938 µg g^−1^.

**Conclusions and Implications:**

This study led to the discovery of REE accumulation in a range of plant species, substantially higher concentrations in species known to be REE hyperaccumulators, and records of REE hyperaccumulators outside of the well-studied populations in China.

## INTRODUCTION

Global herbarium collections are among the most important sources of information for acquiring ionomic, taxonomic, genetic, and biogeographical data in the plant kingdom (Greve *et al.*, 2016; Souza and Hawkins, 2017; Heberling *et al.*, 2019; van der Ent *et al.*, 2019*a*). The term ‘ionome’ refers to the entirety of elements found in plants, including metallic, metalloid and non-metallic elements (Lahner *et al.*, 2003). The terms ‘metallome’ or ‘elementome’ specifically refer to the total composition of metallic or non-metallic elements in a plant (Edwards *et al.*, 2014; Peñuelas *et al.*, 2019). To characterize the complete ionome or metallome of an herbarium plant, a non-destructive analytical method is necessary to preserve the integrity of the specimens. Portable X-ray fluorescence (XRF) spectroscopy fulfils this requirement by emitting high-energy X-rays onto a localized area of the plant (van der Ent *et al.*, 2019*b*). The elements in the radiation zone are excited and in return emit characteristic fluorescent X-rays. Our research builds upon previous studies that utilized portable XRF to obtain elemental data from herbarium specimens (van der Ent *et al.*, 2019*b*; Do *et al.*, 2020; Gei *et al.*, 2020). In our case, the main elements of interest in herbarium plants are the rare earth elements (REEs). Their presence is detected using yttrium (Y) as a proxy for REEs. The choice of Y as a proxy is due to the fact that the most abundant REE in plants [lanthanum (La) and neodymium (Nd)] emit relatively weak L-lines that are indistinguishable from the K-lines produced by transition elements titanium (Ti) to manganese (Mn). Furthermore, the energy generated by the X-ray source (with an Ag-anode) optimally excites the Y Kα-line, producing no interference and allowing very sensitive detection (van der Ent *et al.*, 2023). This approach has been used in a recent study conducted on Queensland herbarium specimens, revealing anomalously high Y concentrations (>1000 µg g^−1^) in the genus *Helicia* (Proteaceae) (Purwadi *et al.*, 2023).

The REEs are a group of 17 chemical elements: 15 lanthanides [atomic numbers (*Z*) from 57 to 71] plus Y (*Z* = 39) and scandium (*Z* = 21). They have similar physical and chemical properties and are typically found in the same minerals. REEs can be divided into two subgroups based on their atomic mass and effective radius: La, Ce, Pr, Nd, Pm, Sm and Eu are classified as light REEs (LREEs), whereas Gd, Tb, Dy, Ho, Er, Tm, Yb, Lu, Sc and Y are classified as heavy REEs (HREEs). REEs are characterized by their low concentration in pure ore deposits and their great importance for modern industry, particularly in energy, telecommunications, electric motors, permanent magnets and lasers (Balaram, 2019). The increasing demand for these metals raises concerns about their environmental and social impact (Rim, 2016). Studies on REE-hyperaccumulating plants are still limited, but it has been shown that such plants may be used to recover these metals from REE mine tailings and REE geochemical anomalous soils (van der Ent *et al.*, 2021).

Hyperaccumulating plants are able to accumulate metallic elements at extremely high concentrations in their leaves (Reeves *et al.*, 1999). Hyperaccumulation is a rare phenomenon that occurs in about 0.2 % of total angiosperms (Reeves *et al.*, 2018). Currently, about 700 hyperaccumulators of various metals have been catalogued in the world. Of these, only 21 (hyper)accumulate REEs (Liu *et al.*, 2021*a*; van der Ent *et al.*, 2021). Several studies have observed correlations between the uptake of REEs and that of macro–micro-elements (Fe, Mg, Ca, Mn, Al, Zn) by plants. One hypothesis explaining this phenomenon is the existence of common pathways for the accumulation of REEs and several essential elements (Grosjean *et al.*, 2020; Liu *et al.*, 2021*a*; van der Ent *et al.*, 2023). The threshold concentration for REE hyperaccumulation is not yet unambiguously defined, but ranges from 100 to 1000 µg g^−1^ (van der Ent *et al.*, 2021). The fern *Dicranopteris linearis* (Gleicheniaceae) has been found in China to accumulate La, Ce, Pr and Nd at 7000 µg g^−1^ in total of dry matter (Shan *et al.*, 2003). In China, the fern species *Blechnopsis orientalis* (as *Blechnum orientale*; Blechnaceae) and *Grypothrix simplex* (as *Pronephrium simplex*; Thelypteridaceae) are known to respectively accumulate 1047 and 3000 µg g^−1^ REEs in their leaves respectively (Lai *et al.*, 2006; Liu *et al.*, 2021*a*; Wang *et al.*, 2023). In the USA, trees of the genus *Carya* (Juglandaceae), with the species *C. tomentosa* and *C. glabra*, have also shown a remarkable ability to accumulate REEs, with total REE concentrations of 1350 µg g^−1^ for the former and 2300 µg g^−1^ for the latter (Robinson *et al.*, 1958; Thomas, 1975). More recently, the herbaceous plant *Phytolacca americana* (Phytolaccaceae), growing naturally near an REE mine in China, had a total leaf concentration of 1040 µg REEs g^−1^ (Yuan *et al.*, 2018).

The objective of this study was to increase the inventory of plant species hyperaccumulating REEs, by identifying new species containing abnormally high concentrations of REEs in their leaves. The portable XRF instrument also allows the concentrations of other elements in the measured specimens to be obtained. Therefore, a secondary objective of this study was to investigate a potential correlation between the foliar accumulation of REEs and foliar accumulation of Mn and Zn. To attain this aim, handheld XRF scanning was performed at the Herbarium of Muséum national d’Histoire naturelle (MNHN, Paris, France).

## MATERIALS AND METHODS

### Selection of herbarium specimens for XRF scanning

To carry out this study, it was necessary to make a preliminary selection based on specimens from the MNHN. This herbarium holds the world’s largest collection of plants, with over 8 million specimens from all five continents (Le Bras *et al.*, 2017). A selection at the family level represented too many specimens to scan, so it was decided to limit the selection to the genus level. In the end, 12 genera representing the two main evolutionary lineages of extant vascular plants (the ferns *Blechnopsis*, *Dicranopteris, Gleichenella* and *Sticherus*; the angiosperms *Annamocarya*, *Carya*, *Engelhardtia*, *Juglans*, *Phytolacca*, *Platycarya*, *Pterocarya* and *Rhoiptelea*), belonging to four plant families (Blechnaceae and Gleicheniaceae in ferns, Juglandaceae and Phytolaccaceae in angiosperms) were selected on the basis of knowledge, giving a total of 4425 specimens analysed during this study. Each specimen is identified with a unique and non-equivocal sample number (PXXXXXXXX), ensuring its traceability and the repeatability of analyses performed at the MNHN.

### Handheld XRF calibration and data processing

A Thermo Fisher Scientific Niton^TM^ XL3t 950 GOLDD+ portable XRF instrument was used in ‘Soils Mode’ coupled with the ‘Main filter’ at 50 kV aiming to excite the K-shell of first-row transition metals. The elements targeted in the specimens analysed by XRF were Y, which served as a tracer for the presence of REEs, as well as Mn and Zn. Each specimen was placed on two pure (99.995 %) 2-mm-thick plates of titanium and molybdenum and measured for 30 s. This setting was used to absorb X-rays transmitted through the specimen and to ensure a uniform background. Each specimen was measured once only at leaf laminae. This procedure produces errors of less than 4 % compared to the mean concentration of the whole leaf for the first-row transition metals (Purwadi *et al.*, 2022). The obtained spectra were processed in GeoPIXE 7.5, a software package based on Dynamic Analysis that has been developed for synchrotron-based XRF and nuclear microprobe techniques (Ryan, 2000; Ryan *et al.*, 2005). The instrument was calibrated in a previous study (Purwadi *et al.*, 2022) to obtain relevant instrumental parameters (filter material and thickness, source composition, detector dimensions, etc.) required by GeoPIXE. The Dynamic Analysis method is a Fundamental Parameter approach that solves complex physics equations (Sherman, 1955) by iteratively fitting linear and non-linear models to decompose the full XRF spectrum to a single spectrum of each element within the sample that contributes to the full XRF spectrum (Ryan, 2000; Ryan *et al.*, 2005, 2015). The LODs for these elements, determined by GeoPIXE analysis of sample spectra, ranged from 49 to 73 µg g^−1^ for Y, 115 to 165 µg g^−1^ for Mn, and 77 to 113 µg g^−1^ for Zn. For all three elements, concentrations >LOD were observed in each family. Taking the prevailing Y concentrations in *D. linearis* (Wei *et al.*, 2005; Liu *et al.*, 2020; van der Ent *et al.*, 2023), *Helicia* species (herbarium specimens) (van der Ent *et al.*, 2023) and *Carya* species (Wood and Grauke 2011) compared to tREE (totalREEs) yields a range of 1.3–20.2 % of the total tREEs, and taking the mean Y (9.9 %) and established tREE hyperaccumulation threshold (1000 µg g^−1^) this translates to a Y hyperaccumulation threshold of 99 µg g^−1^. Given the uncertainty about the REE hyperaccumulation phenomenon, considering normal background concentrations of <5 µg g^−1^ Y and to avoid missing potential REE hyperaccumulators with a low Y to REE ratio, we take half this value and refer to plants as Y hyperaccumulators if exceeding 50 µg g^−1^ Y.

### Destructive sample analysis with ICP-AES

Analyses were performed on ten specimens identified with high foliar concentrations of Y based on XRF analysis (*Blechnopsis orientalis n* = 4; *Dicranopteris linearis n* = 3; *Dicranopteris flexuosa n* = 2; *Gleichenella pectinata n* = 1). The measured samples represent a leaf section of ~2 cm^2^ from the identified specimens. They were finely ground (<250 µm) and weighed at 0.05 g (± 0.005 g), except for samples P01315862, P01571495, P01315958 and P01474002 where the plant material was insufficient. The dried plant material was digested in an HNO_3_ + H_2_O_2_ mixture (1:3, v/v) for 16 h at room temperature before heating at 95 °C for 2 h in a graphite heating block (DigiPREP® system, SCP Science, Baie-d’Urfé, QC, Canada). After filtration at 0.45 µm, the plant samples were analysed using induced coupled plasma atomic emission spectrometry (ICP-AES, iCAP 6000 series, Thermo Scientific, Cambridge, UK) to measure the concentrations of REEs and other elements such as as Mn and Zn. For quality control, a certified reference sample (BCR-670, *Lemna minor*) and an internal control sample (LSE-EchCont-20, *Noccaea caerulescens*) with known values were used.

### Synchrotron µXRF experiments

The XRF microscopy experiments were undertaken at PETRA III (Deutsches Elektronen-Synchrotron DESY), a 6-GeV synchrotron radiation source, specifically at the hard X-ray microprobe undulator beamline P06 (Boesenberg *et al.*, 2016). P06 is equipped with a cryogenically cooled double-crystal monochromator with Si(111) crystals. Using different focusing optics, the X-ray beam can be focused down to sub-micrometre level. An ion chamber upstream of the sample is used to monitor the incoming flux, while a 500-µm-thick Si PIPS diode with a 19-mm-diameter active area [PD300-500CB, Mirion Technologies (Canberra) GmbH, Germany] located downstream of the sample can be used to record the transmitted X-ray intensity in order to extract absorption data. Multiple XRF detectors allow for the measurement of XRF data. The incident X-ray energy was 18 keV for the whole experiment and focused with K-B mirrors to 3.57 × 0.92 µm (h × v), resulting in a flux of about 1.25^11^ photons s^–1^ in the focus. For XRF detection, both a Vortex ME4 in ~45° geometry with detector–sample distance ~5.5 cm and a prototype 16-element SDD Ardesia detector (800-µm-thick chip with about 324-mm^2^ combined active area for all 16 elements, Politecnico Milano, Italy; Utica *et al.*, 2021) in 315° geometry with detector–sample distance ~6.0 cm with Xspress 3 pulse processors were used.

### Data presentation and analysis

The elemental concentrations reported by GeoPIXE were processed further in R v4.1.1 and RStudio v1.4.1106. Dunn’s Kruskal–Wallis post-hoc test was performed to check the similarity in elemental distribution across the family by using the ‘FSA’ package (P ≤ 0.05). For data analysis, all graphs in this study were created using RStudio v4.0.2 and the ‘ggplot2’ package. Violin plots were generated using analytical data that included only specimens with detection values for the elements of interest (Y, Mn, Zn) above the limits of detection (>LOD). The same data were also used to create vertical and horizontal bar plots. The *x*-axis scale of the vertical bar plots was transformed to log_10_. The skewness of the distributions was assessed using the skewness test from the ‘PerformanceAnalytics’ package in Rstudio. Hartigan’s dip test was performed for each vertical bar plot, except for Y with the Juglandaceae (*n* = 5) and Phytolaccaceae (*n* = 6) families due to an insufficient number of values. This test was used to assay the unimodality in the frequency distributions with the ‘dip test’ package in RStudio. The horizontal bar plots show the number of analysed specimens belonging to different accumulation levels (normal, accumulator and hyperaccumulator) for Y, Mn and Zn defined from the literature (Table 1). The heatmap was generated based only on species with at least one specimen with a Y-value >LOD. The percentages shown indicate the proportion of specimens with detection values >LOD compared to the total number of specimens analysed in this study for a given species and a given element of interest (Y, Mn, Zn). To validate the method used in this study, a linear regression was applied to compare the Y concentrations obtained by ICP-AES with those obtained by the handheld XRF detector. To this end, ten samples measures with handheld XRF device with low, medium and high Y concentrations were selected and destructively sampled (Supplementary Data Fig. S1). Each specific sample was first measured twice with the handheld XRF, and acid digested and analysed by ICP-AES. A Pearson test was carried out confirming a positive correlation between these two modes of measurement (Pearson correlation 0.84, *P* = 0.0022). The synchrotron micro-XRF (µXRF) event stream was processed using non-linear least-squares fitting as implemented in PyMCA (Solé *et al.*, 2007). Figures were prepared in ImageJ (Schneider *et al.*, 2012) by changing the LUT (Look up Table) to ‘Fire’ and adjusting of the maximum values and adding length scales.

**Table 1.** Concentrations of Y, Mn and Zn relative to their different accumulation classes and the LOD values for these elements with the XRF instrument.

Class	Y (µg g^−1^)	Mn (µg g^−1^)	Zn (µg g^−1^)
Normal concentrations	<5	50–500	50–200
Accumulator	5–50	500–3000	200–3000
Hyperaccumulator	>50	>3000	>3000
LOD of XRF method	49–73	115–165	77–113

## RESULTS

### Overall observations on the handheld XRF dataset

This study analysed 142 species (Supplementary Data Table S1) from 12 genera, belonging to four plant families, for a total of 4425 scanned specimens. It should be noted that this study did not examine all the genera of each family mentioned above, but only the 12 genera mentioned. Hence, the results presented at the family level were not exhaustive for these families but referred only to specimens belonging to the specific genera. However, all specimens belonging to these genera and present at the MNHN in Paris were analysed using handheld XRF, except for the genus *Blechnopsis*, for which only one species, *B. orientalis*, was analysed. The elements of interest in this study were Y, Mn and Zn. Y was detected in a total of 268 specimens (6.1 % of the total specimens scanned) belonging to 16 species and seven genera (Table 2). Mn concentrations >LOD was found in 1440 specimens (32.5 %) belonging to 97 species and 12 genera, and 345 specimens (7.8 %) belonging to 64 species and nine genera had Zn concentrations >LOD.

**Table 2.** Total number of specimens scanned and percentage of specimens above the XRF detection limits of Y, Mn and Zn for each family.

	Specimens scanned	Y		Mn		Zn	
Blechnaceae	561	149	27 %	27	5 %	20	4 %
Gleicheniaceae	1310	108	8 %	477	36 %	52	4 %
Juglandaceae	1417	5	0.3 %	582	41 %	125	9 %
Phytolaccaceae	1137	6	0.5 %	354	31 %	148	13 %
Total	4425	268	6.1 %	1440	32.5 %	345	7.8 %

### General description of the distribution of Y, Mn and Zn concentrations among families

**Fig. 1.**
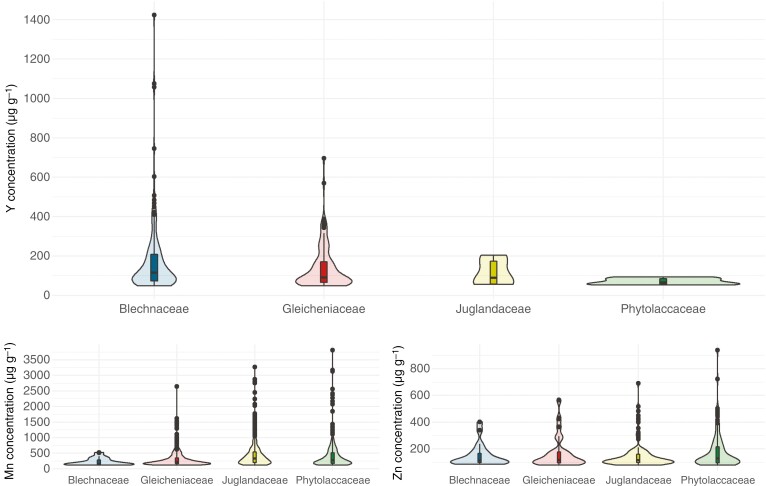
Violin plots showing the distribution of foliar concentrations of Y, Mn and Zn obtained with the XRF instrument within the families Blechnaceae, Gleicheniaceae, Juglandaceae and Phytolaccaceae.

Violin plots show the distribution of specimens according to their concentrations of elements of interest (Y, Mn, Zn) based on the family to which they belong (Fig. 1). Among the four families studied, Blechnaceae represented 56 % (*n* = 149) of the total number of specimens with leaf Y concentrations >LOD. The foliar Y concentrations measured in this family were the most dispersed with values ranging from 49 to 1424 µg g^−1^. A lower dispersion was found in the Gleicheniaceae, for which 108 specimens (40 %) were found with foliar Y concentrations ranging from 49 to 697 µg g^−1^. In the other two families, only five specimens (2 %) from the Juglandaceae had foliar Y concentrations ranging from 56 to 205 µg g^−1^, and six specimens (2 %) from the Phytolaccaceae had foliar Y concentrations ranging from 59 to 94 µg g^−1^. The distribution of Y concentrations observed among families is related to the relatively high LOD value of Y with the handheld XRF method (49 µg g^−1^) and only specimens with exceptionally high Y concentrations could be identified here.

For Mn and Zn, the four families also showed different dispersion profiles, but with an opposite pattern. Thus, the Phytolaccaceae showed the greatest variability for both elements. For Mn, concentrations ranged from 116 to 3807 µg g^−1^ in a total of 354 specimens (25 %), and for Zn concentrations ranged from 78 to 938 µg g^−1^ in a total of 148 specimens (43 %). The dispersion was slightly lower in the Juglandaceae. Of a total of 582 specimens (40 %), Mn concentrations ranged from 116 to 3265 µg g^−1^. For Zn, 125 specimens (36 %) were measured with foliar concentrations ranging from 77 to 691 µg g^−1^. With a similar profile, the Gleicheniaceae had a lower variability. Of a total of 477 specimens (33 %), Mn concentrations ranged from 116 to 2644 µg g^−1^. For Zn, concentrations ranged from 77 to 567 µg g^−1^ in a total of 52 specimens (15 %). Finally, the Blechnaceae had the lowest variability with 27 specimens (2 %) with Mn concentrations ranging from 121 to 522 µg g^−1^ and 20 specimens (6 %) with Zn concentrations ranging from 84 to 402 µg g^−1^.

### (Hyper)accumulation potential of Y, Mn and Zn at the family level

**Fig. 2.**
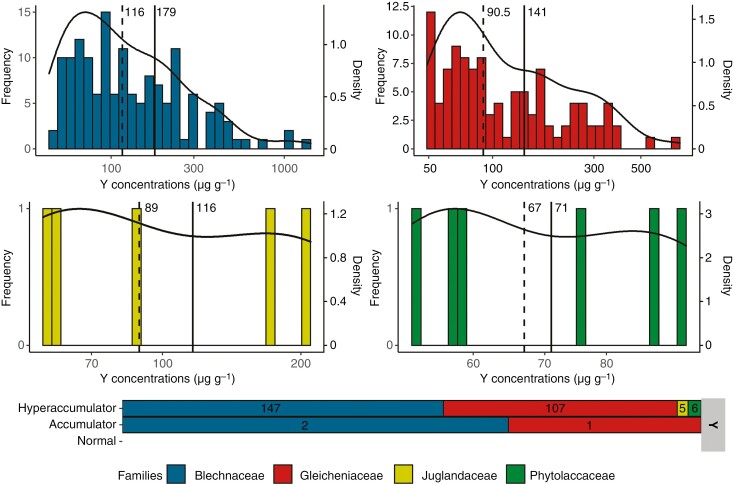
(A) Distribution of specimens by foliar concentrations of Y for Blechnaceae, Gleicheniaceae, Juglandaceae and Phytolaccaceae. Solid lines correspond to mean values and dashed lines to median values. The specimens shown are only those analysed with a Y concentration >LOD. Black curves represent the density curves of the distributions. (B) Number of specimens belonging to different accumulation levels of Y (Table 1).

The frequency distribution and the density curve for Y concentrations in the herbarium specimens (Fig. 2A) were positively skewed for the families Blechnaceae and Gleicheniaceae with a tail spreading to the right. For both families, the majority of specimens with Y values >LOD (69 and 66 %, respectively) had concentrations below their mean concentrations (179 and 141 µg g^−1^, respectively). Skewness tests confirmed the positive skewness of both distributions (2.16 and 3.61) but with a unimodality of both datasets confirmed by Hartigans’ dip tests (*P* = 0.75 and *P* = 0.95). In total, 147 plant specimens from the Blechnaceae and 107 plant specimens from the Gleicheniaceae had Y foliar concentrations >50 µg g^−1^, making them Y hyperaccumulators (Fig. 2B). The numbers of specimens from the Juglandaceae and Phytolaccaceae with Y values >LOD were too low to identify a trend in the frequency distribution of Y concentrations. However, all of these specimens, five and six respectively, may be considered as hyperaccumulators.

**Fig. 3.**
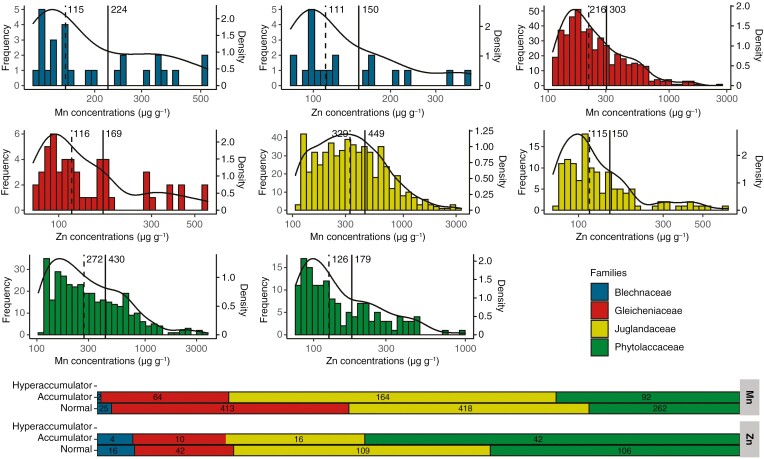
(A) Distribution of specimens by foliar concentrations of Mn and Zn for Blechnaceae, Gleicheniaceae, Juglandaceae and Phytolaccaceae. Solid lines correspond to mean values and dashed lines to median values. The specimens shown are only those analysed with an Mn concentration >LOD and a Zn concentration >LOD. Black curves represent the density curves of the distributions. (B) Number of specimens belonging to different accumulation levels of Mn and Zn (Table 1).

The frequency distributions of Mn and Zn concentrations in the herbarium specimens were positively skewed with a tail spreading to the right for the four families studied (Fig. 3A). This was confirmed by skewness tests which were all significantly greater than 1 for both Mn and Zn and the four families. These distributions were also unimodal, which were confirmed by Hartigans’ dip tests whose *P*-values were much greater than 0.05 for each distribution. Of the 1440 specimens with Mn values >LOD, 322 were between 500 and 3000 µg g^−1^ and considered as Mn accumulators. This included two specimens from the Blechnaceae, 64 from the Gleicheniaceae, 164 from the Juglandaceae and 92 from the Phytolaccaceae (Fig. 3B). None of the scanned specimens were above the hyperaccumulation threshold for Mn (3000 µg g^−1^). Finally, of the 345 specimens with Zn values >LOD, 72 were between 200 µg g^−1^ and 3000 µg g^−1^ and considered as Zn accumulators. This included four specimens from the Blechnaceae, 14 from the Gleicheniaceae, 16 from the Juglandaceae and 42 from the Phytolaccaceae (Fig. 3B).

### (Hyper)accumulation potential of Y, Mn and Zn at the species level

**Fig. 4.**
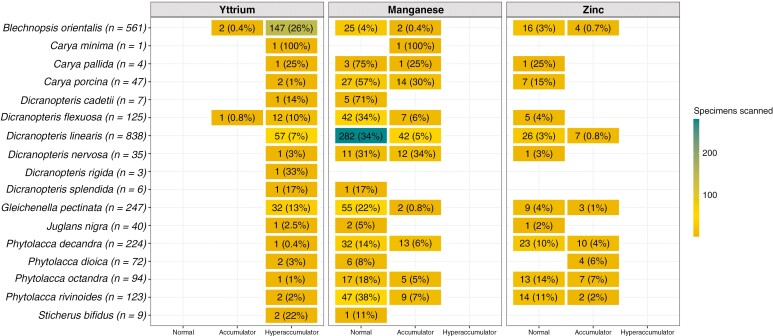
Species-level representation of the number of specimens analysed with foliar concentrations of Y, Mn and Zn >LODs. Only species containing at least one specimen with a foliar concentration of Y >LOD are shown. Percentages are calculated relative to the total number of specimens analysed for a given species. The accumulation categories refer to those in Table 1.

Among the 12 plant genera analysed in this study, seven contained a total of 268 specimens with Y concentrations >LOD in their leaves: *Blechnopsis* (*n* = 149), *Dicranopteris* (*n* = 75), *Gleichenella* (*n* = 32), *Phytolacca* (*n* = 6), *Carya* (*n* = 4), *Juglans* (*n* = 1) and *Sticherus* (*n* = 1) (Fig. 4). Of these specimens, 265 were hyperaccumulators of Y and were distributed among 16 species as shown in Fig. 4. In the ‘hyperaccumulator’ category, *B. orientalis* comprised 147 specimens (26 % of the specimens scanned for this species) of the genus *Blechnopsis*, noting that *B. orientalis* was the only species (out of two recognized) scanned for this genus. For *Dicranopteris*, 57 specimens (7 %) belonged to *D. linearis* and 13 (10 %) to *D. flexuosa* and for *Gleichenella* 32 (13 %) belonged to *G. pectinate*. The remaining specimens (*n* = 7) were distributed among the following species: *S. bifidus*, *D. cadetii*, *D. nervosa* and *D. splendida*. For *Phytolacca*, the six specimens hyperaccumulating Y were distributed among the four species *P. decandra*, *P. dioica*, *P. octandra* and *P. rivinoides*. For *Carya*, the four specimens were distributed among *C. minima*, *C. pallida* and *C. porcina*. Finally, one specimen of *Juglans nigra* was identified as a Y hyperaccumulator. Among the 16 species with foliar Y concentrations >LOD, 556 and 108 specimens were found respectively as normal and accumulator plants regarding their foliar Mn concentrations. These latter were distributed in five genera: *Dicranopteris* (*n* = 61), *Phytolacca* (*n* = 27), *Carya* (*n* = 16), *Gleichenella* (*n* = 2) and *Blechnopsis* (*n* = 2). Finally, for Zn and still on the basis of the 16 species mentioned above, 116 plant specimens had ‘normal’ foliar Zn concentrations, and 37 were identified as Zn accumulators. These 37 specimens were distributed in four genera: *Phytolacca* (*n* = 23), *Dicranopteris* (*n* = 7), *Blechnopsis* (*n* = 4) and *Gleichenella* (*n* = 3).

### Focus on high range Y species (B. orientalis, D. linearis, D. flexuosa and G. pectinata)

The concentrations of REEs and other elements obtained by ICP-AES for *B. orientalis* (*n* = 4), *D. linearis* (*n* = 3), *D. flexuosa* (*n* = 2) and *G. pectinata* (*n* = 1) are shown in Supplementary Tables S2 and Table 3. The highest concentration of REEs was found in *D. linearis*, with a total of 6423 µg g^−1^. In *B. orientalis*, all four samples had REE concentrations >2000 µg g^−1^, with a maximum of 4278 µg g^−1^. Finally, 2780 and 1447 µg g^−1^ of REEs were found in *D. flexuosa* and *G. pectinata*, respectively. Yttrium was the most abundant REE in these ten plant samples (24 %), and LREEs were present in much higher concentrations than HREEs.

**Table 3.** Concentrations of other elements in the samples analysed by ICP-AES. The concentrations are given in µg g^−1^ dry matter.

Family	Species	Specimen ID (barcode of the Paris Herbarium sheet)	Ca	Fe	K	Mn	P	Zn
Blechnaceae	*B. orientalis*	P01576169	4649	1592	10 769	35	595	45
		P01618486	6046	2159	6609	20	710	54
		P01571495	6431	1812	10 544	54	577	50
		P01619249	4080	1497	3956	66	1105	107
Gleicheniaceae	*D. flexuosa*	P01315784	3390	356	4947	198	229	37
		P01315862	669	15 467	6714	70	605	92
	*D. linearis*	P01315958	4142	5404	8396	773	964	224
		P01474002	3501	3511	4915	1051	1500	325
		P00139656	1142	2415	6084	270	605	206
	*G. pectinata*	P01316111	676	953	7850	69	420	29

### Synchrotron µXRF analysis

**Fig. 5.**
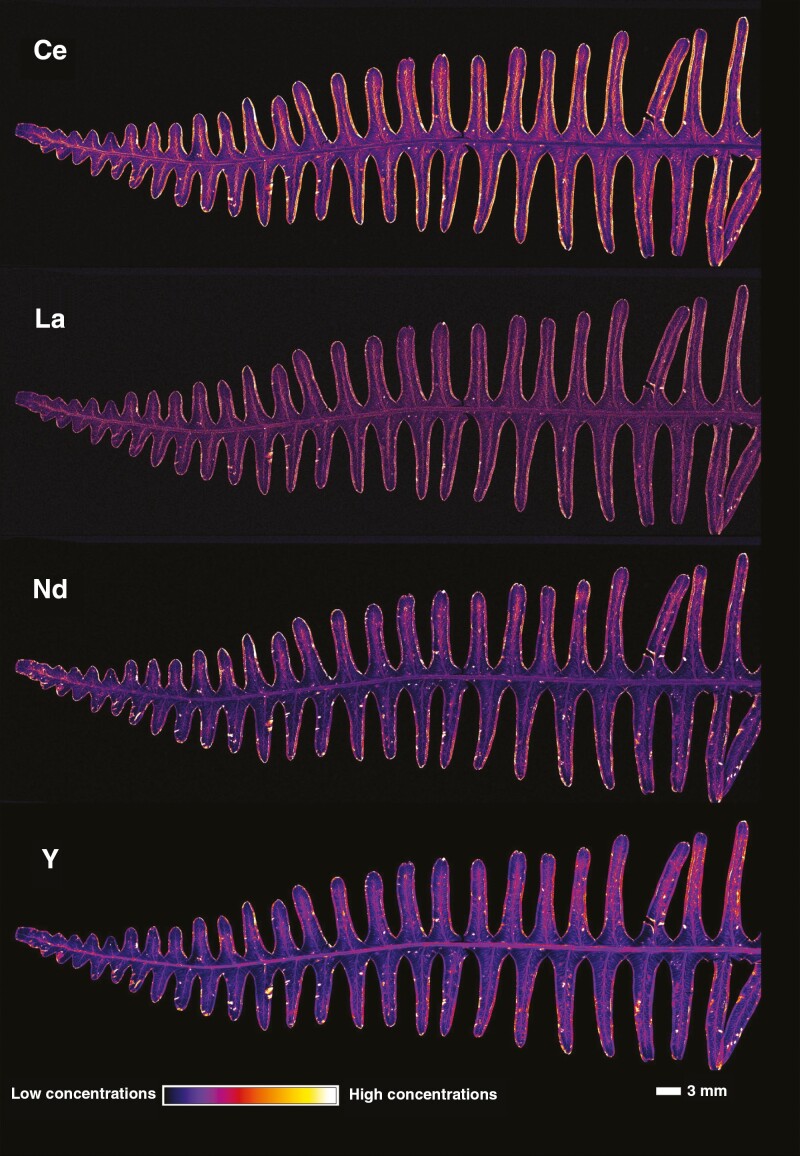
Synchrotron µXRF elemental maps showing the distributions of Ce, La, Nd and Y in a frond of *Dicranopteris linearis* (specimen P01523962). The scan measures 91.33 × 32 mm, with a 20-µm step size and 5-ms dwell time.

**Fig. 6.**
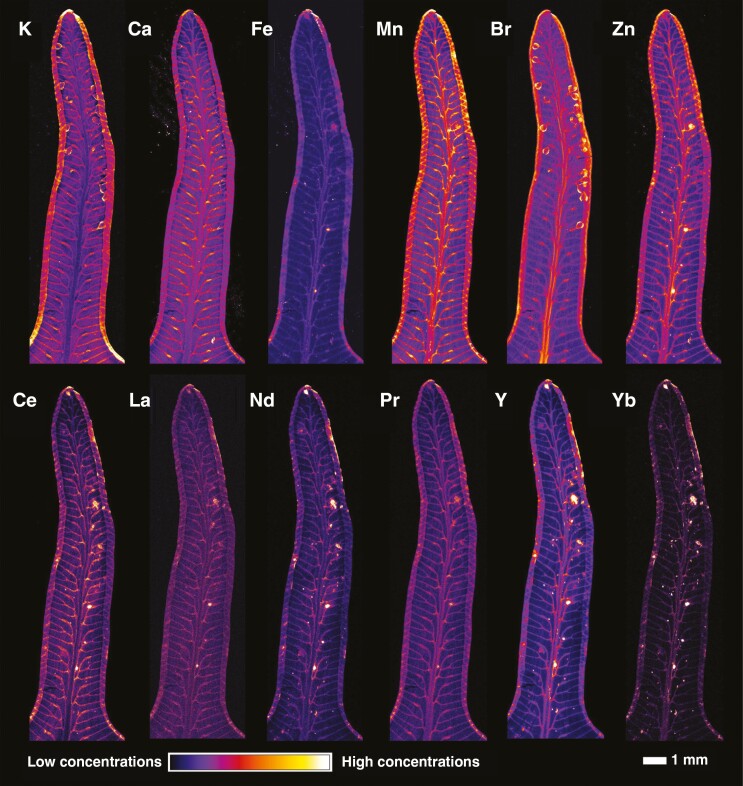
Synchrotron µXRF elemental maps showing the distributions of K, Ca, Fe, Mn, Br, Zn, Ce, La, Nd, Pr, Y and Yb in a pinnule of *Dicranopteris linearis* (specimen P01523962). The scan measures 15.88 × 4.15 mm, with a 6-µm step size and 5-ms dwell time.

Synchrotron µXRF was used to investigate whether there was any surgical contamination on the specimens that could have influenced the results of the handheld XRF scanning, and to provide information about the distribution of the REEs in the tissues. For this, a specimen (P01523962) with high Y concentration (1032 µg g^−1^ by handheld XRF) was selected and a 10-cm frond fragment extracted. An overview scan (Fig. 5) revealed that Ce concentrations in the vascular bundles of the pinnules and the distributions of La and Nd are similar, whilst Y is also enriched in areas surrounding the veins. There is notable enrichment in the margins of the pines, but this probably results from curling of the pinnule edges. A high-resolution scan of a single pinnule (Fig. 6) provides further detail on these distributions and shows that the REEs (Ce, La, N, Pr) have similar distributions with localization in the secondary vascular bundles and some minor (necrotic) hotspots. The distributions of light (K, Ca) and transition elements (Fe, Mn, Zn) as well of those of Br and Rb are also provided (Fig. 6; Supplementary Data Figs S2 and S3). Very low Fe concentrations and absence of hotspot specks (e.g. soil particles) strongly suggest that surgical contamination is negligible. The distribution of Br is concentrated in sporangia in the pinnule.

## DISCUSSION

Herbarium XRF scanning is an effective method that can be used to discover new metal/metalloid (hyper)accumulator plant species (Nkrumah *et al.*, 2018; van der Ent *et al.*, 2019*a*; Gei *et al.*, 2020). This approach allowed us to measure 4425 specimens held in the MNHN in Paris in search of novel REE (hyper)accumulators and led to amongst the highest values for REE hyperaccumulation in plants: *D. linearis* at 6423 µg g^−1^. In this study, for methodological reasons, Y was used as a proxy for REEs. For example, Y represents 6 % of the total REEs accumulated by *D. linearis* growing on ion adsorption REE mine tailings in China (Liu *et al.*, 2020) and 24 % of the total REEs present in the ten plant samples analysed by ICP-AES in this study.

The XRF scanning results show that 147 specimens of *B. orientalis* and 56 specimens of *D. linearis* accumulate Y in their leaves up to concentrations of 1424 and 697 µg g^−1^, respectively. The fern *D. linearis* is widely documented for its REE (hyper)accumulation capabilities in China, but is in fact polyphyletic, representing a species complex of cryptic species (Wei *et al.*, 2022; Vieira Lima *et al.*, 2023). This study reveals that some specimens of *D. linearis* accumulating >1000 µg g^−1^ REEs in their fronds are from Indonesia and the Philippines, but these populations may well represent distinct species within the *D. linearis* complex. Although much less studied than *D. linearis*, another fern from a different family (Blechnaceae), *B. orientalis*, has been shown by Zhang *et al.* (2004) and Wang *et al.* (2023) to be capable of accumulating up to 865–1047 µg g^−1^ total REEs. The present study shows that a significant proportion of the analysed *B. orientalis* specimens accumulate high concentrations of Y, as confirmed by ICP-AES analyses with a concentration of total REEs reaching 4278 µg g^−1^ in a specimen. Therefore, further studies on the potential of *B. orientalis* as an REE hyperaccumulator should be conducted to confirm its REE accumulation capabilities and determine its bioconcentration factor. Furthermore, this study shows that *B. orientalis* and *D. linearis* have accumulated high concentrations of REEs outside of the known REE ion exchange deposits in China. This is relevant because REE hyperaccumulation has previously mainly been identified in these REE-enriched areas. Discovery of REE hyperaccumulators in Indonesia and the Philippines may lead to the discovery of new deposits in these countries, by using them as indicators for REE deposits.

Previous research has identified correlations between the uptake of REEs by plants and that of certain micronutrients. For instance, Wood and Grauke (2011) reported a positive relationship between the accumulation of Mg, Ca, Mn and Fe and the foliar concentrations of REEs in *Carya*. Similarly, Liu *et al.* (2021*b*) found a positive association between the accumulation of REEs and foliar concentrations of Al, Fe and Zn in *Phytolacca americana*. In several fern genera, such as those examined by Grosjean *et al.* (2020), foliar REE concentrations were also positively correlated with the uptake of multiple microelements. In our study, however, we did not observe any correlation between Y and Mn or Zn; only 18 specimens accumulated Y and Mn and six accumulated Y and Zn, which may be due to differences in the leaf distribution of these elements as well as to the high LOD values of our instrument.

Furthermore, this study led to the discovery of 15 additional plant species that are potential REE hyperaccumulators, namely the trees *Carya minima*, *C. pallida*, *C. porcina* and *Juglans nigra*; the ferns *Gleichenella pectinata*, *Dicranopteris cadetti*, *D. flexuosa*, *D. nervosa*, *D. splendida* and *Sticherus bifudus*; and the dicot herbs *Phytolacca decandra*, *P. dioica*, *P. octandra* and *P. rivinoides*. Surprisingly, no specimens of *Carya tomentosa* or *Phytolacca americana* were identified with foliar Y concentrations >LOD, even though these species are known to accumulate >1000 µg g^−1^ REEs in their leaves (Thomas, 1975; Liu *et al.*, 2020). However, the minimum concentrations obtained in this study are limited by the LOD value of Y (47 µg g^−1^). This value is much higher than the concentrations normally present in plants, which results in exceptional specimens being highlighted at the expense of normal and abnormal specimens.

## CONCLUSIONS

REE hyperaccumulation is a widespread phenomenon in spore-bearing vascular plant (‘Pteridophytes’) species. To date, no genus showing REE hyperaccumulation has been found in the lycophytes, but 20 genera of ferns have been identified with species that can accumulate REEs in their leaves: *Adiantum*, *Alsophila*, *Asplenium*, *Athyrium*, *Austroblechnum* and *Blechnopsis* (analysed as *Blechnum* spp. *s.l*. in previous studies), *Cystopteris*, *Christella* (as *Cyclosorus*), *Dicranopteris*, *Diplazium*, *Dryopteris*, *Gleichenella*, *Gleichenia*, *Grypothrix* (as *Pronephrium*), *Odontosoria* (as *Stenoloma*), *Onoclea*, *Polystichum*, *Pteridium*, *Sticherus* and *Woodwardia* (Ichihashi *et al.*, 1992; Ozaki *et al.*, 2000; Zhang *et al.*, 2004; Lai *et al.*, 2005; Grosjean *et al.*, 2020; Chen *et al.*, 2022). Therefore, it appears that ferns possess a trait that allows them to accumulate high concentrations of REEs. The reasons for this phenomenon are not yet known, but one possibility is that it allows ferns to have greater resilience in their environment (Grosjean *et al.*, 2020) even though many other ferns are considered as bioindicators as they are susceptible to environmental change (Silva *et al.*, 2018). Moreover, among the 22 *Dicranopteris* species analysed in the present study, seven in addition to *D. linearis* could be potential REE accumulators. Wood and Grauke (2011) reported the presence of a trait for REE accumulation in *Carya* species, which all accumulate REEs in their leaves. Grosjean *et al.* (2020) reported high REE accumulation in *Athyrium* species, unlike *Polypodium* species, which are lower level accumulators. The conservation of this trait for REE accumulation within ‘Pteridophyte’ genera could lead to the discovery of numerous additional REE (hyper)accumulating species. The usefulness of Y as an indicator for REEs in XRF analysis of herbarium specimens has been demonstrated in this study.

## SUPPLEMENTARY DATA

Supplementary data are available at *Annals of Botany* online and consist of the following.

Figure S1. Regression line between Y concentrations measured by handheld XRF and subsequently analysed by ICP-AES after acid digestion. Figure S2. Synchrotron µXRF elemental maps showing the distributions of K, Ca, Mn and Zn in a frond of *Dicranopteris linearis* (specimen P01523962). Figure S3. Synchrotron µXRF elemental maps showing the distributions of Cu, Br, Fe and Rb in a frond of *Dicranopteris linearis* (specimen P01523962). Table S1. Number of specimens scanned by species. Table S2. Concentrations of the different REEs in the samples analysed by ICP-AES. The concentrations are given in µg g^1^ of dry matter. LQ, limit of quantification of the ICP-AES instrument.

mcae011_suppl_Supplementary_Figures_S1-S3_Table_S1

mcae011_suppl_Supplementary_Table_S2

## Data Availability

The data that support this study will be shared upon reasonable request to the corresponding author.
