# Supplementary material for: Prospecting for rare earth element (hyper)accumulators in the Paris Herbarium using X-ray fluorescence spectroscopy reveals new distributional and taxon discoveries
Source: Ann Bot. 2024 Feb 3;133(4):573–84. doi: 10.1093/aob/mcae011 (PMC11037481; doi:10.1093/aob/mcae011)
Supplement: mcae011_suppl_Supplementary_Figures_S1-S3_Table_S1 [file mcae011_suppl_supplementary_figures_s1-s3_table_s1.pdf]

SUPPLEMENTARY INFORMATION

Prospecting for rare earth element (hyper)accumulators in the Paris Herbarium using X-ray fluorescence spectroscopy reveals new distributional and taxon discoveries

*Léo Goudard[1], Damien Blaudez[2], Catherine Sirguey[1], Imam Purwadi[3], Vanessa Invernon[4], Germinal Rouhan[4], Antony van der Ent[1,3,5]**

[1]Université de Lorraine, INRAE, LSE, F-54000, Nancy, France

[2]Université de Lorraine, CNRS, LIEC, F-54000, Nancy, France

[3]Centre for Mined Land Rehabilitation, Sustainable Minerals Institute, The University of Queensland, Brisbane, Australia

[4]Institut de Systématique, Evolution, Biodiversité (ISYEB), Muséum national d'Histoire naturelle, CNRS, Sorbonne Université, École Pratique des Hautes Études, Université des Antilles, Paris, France

[5]Laboratory of Genetics, Wageningen University and Research, Wageningen, The Netherlands

* For correspondence. E-mail antony.vanderent@wur.nl

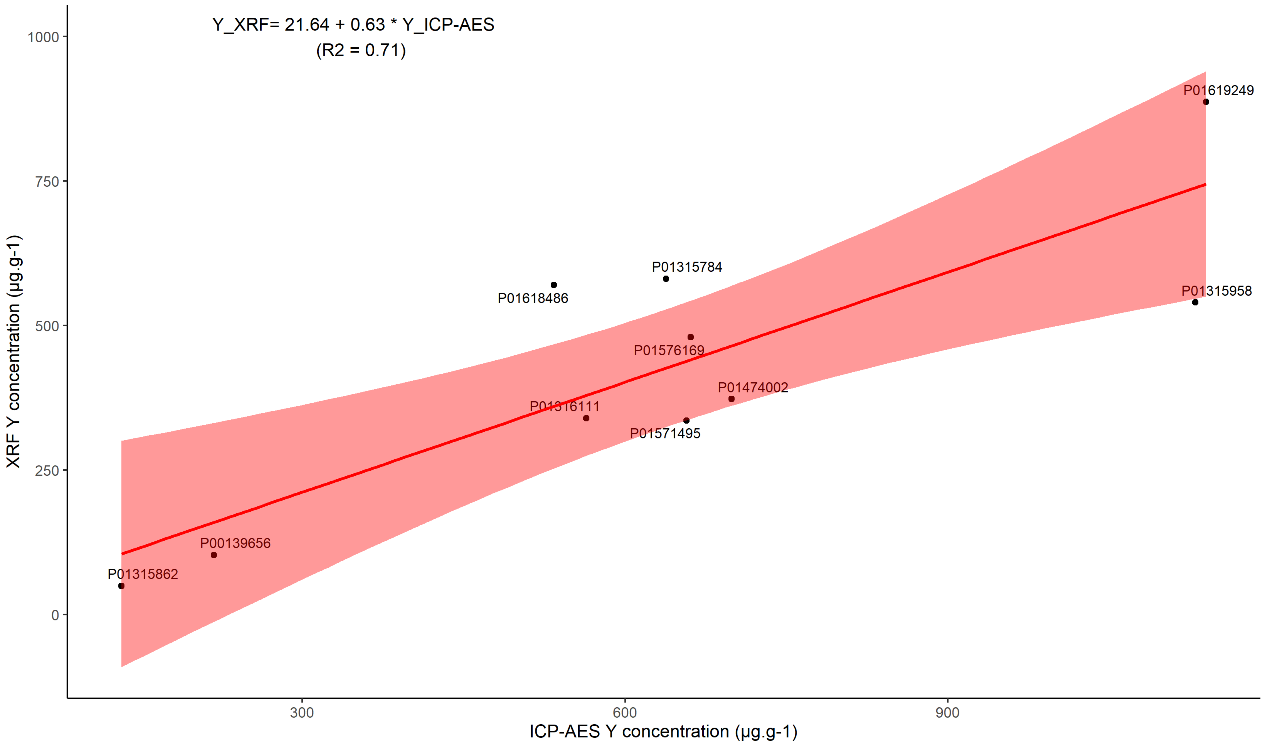

Suppl Fig 1. Regression line between the Y concentrations measured by handheld XRF and subsequently analysed by ICP-AES after acid digestion; herbarium barcodes of the Paris herbarium sheets of samples are indicated.

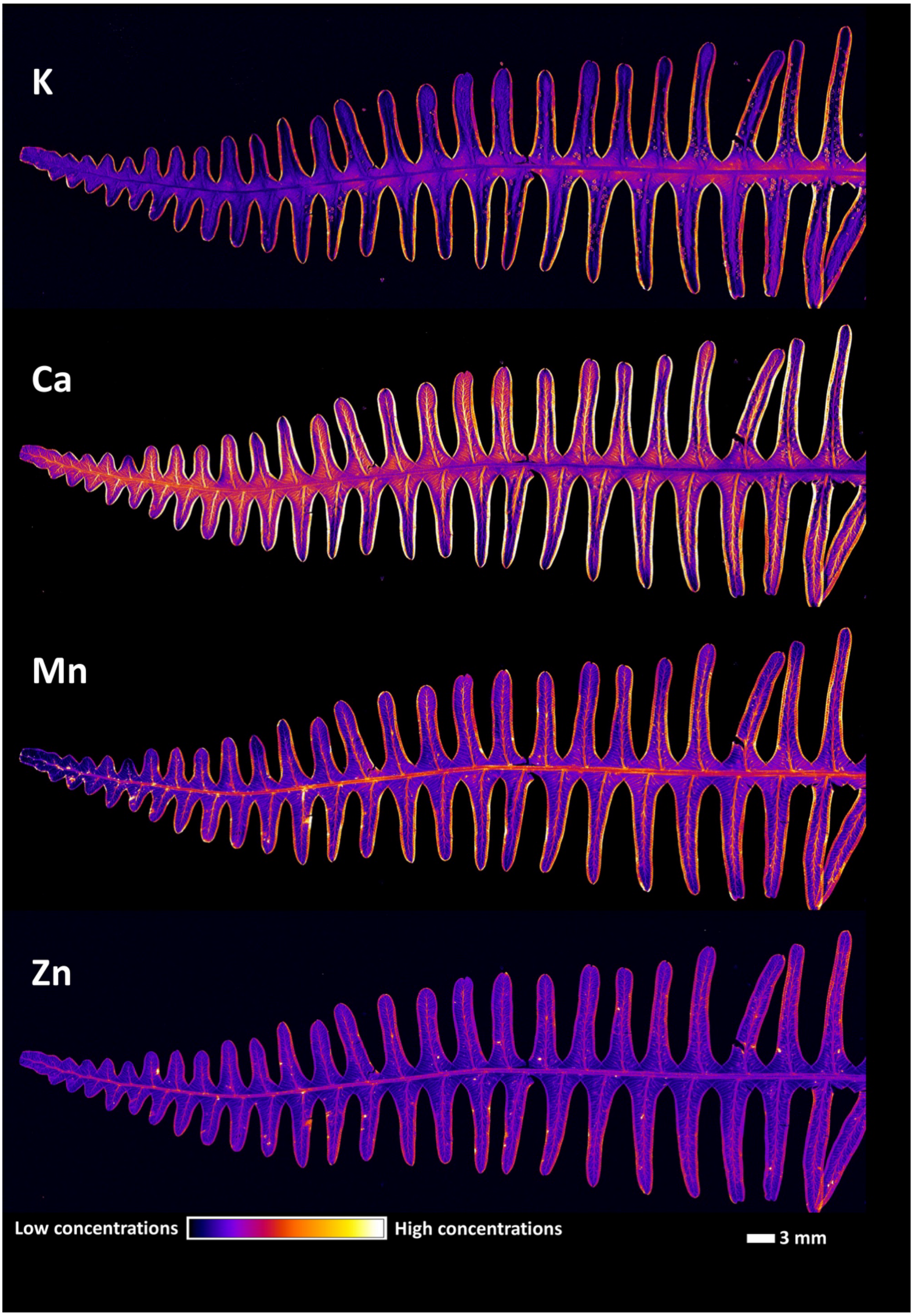

Suppl Fig 2. Synchrotron µXRF elemental maps showing the distributions of K, Ca, Mn and Zn in a frond of *Dicranopteris linearis* (specimen P01523962).

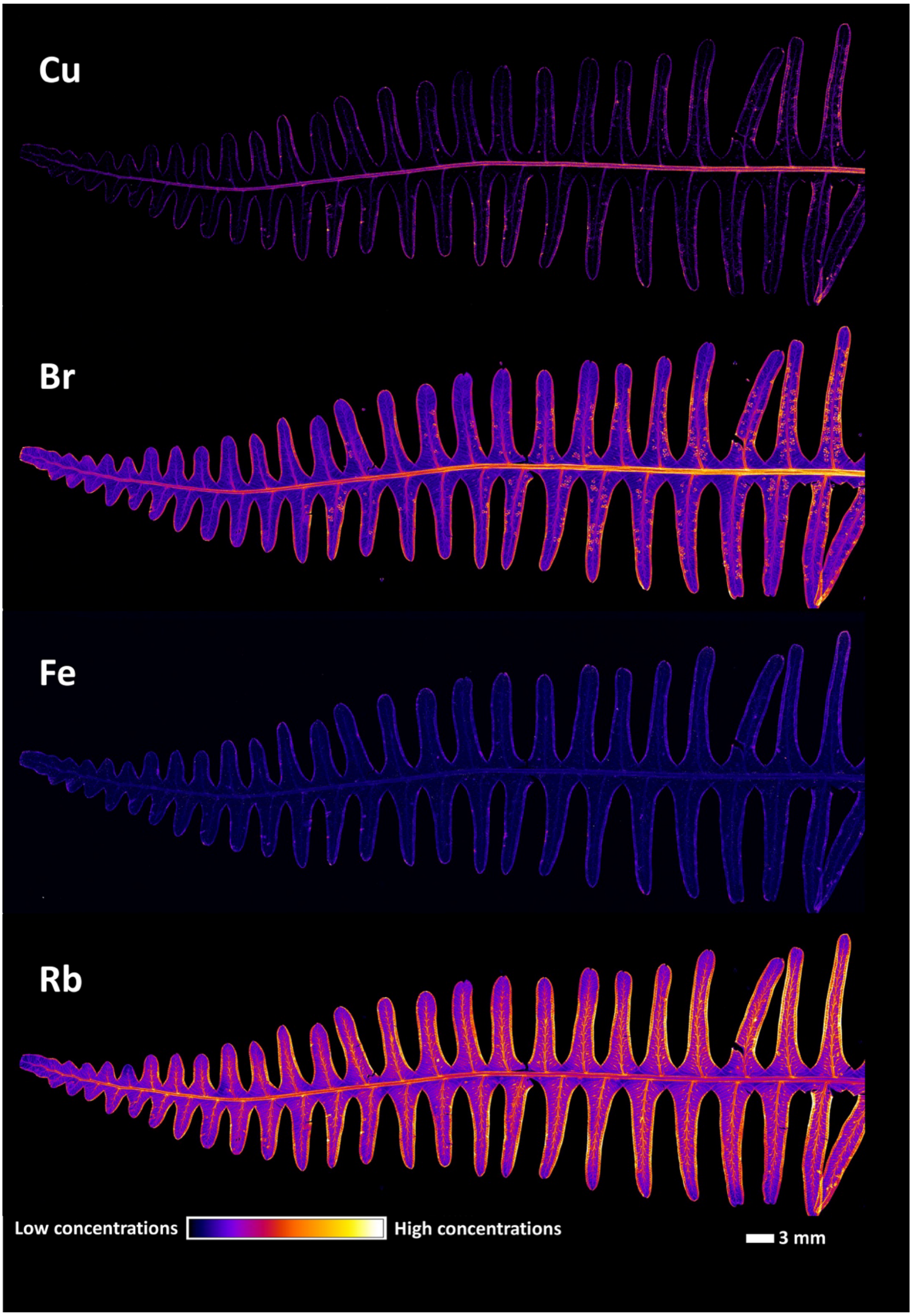

Suppl Fig 3. Synchrotron µXRF elemental maps showing the distributions of Cu, Br, Fe and Rb in a frond of *Dicranopteris linearis* (specimen P01523962).

Suppl Table 1. Number of specimens scanned by species.

FAMILY	GENUS	SPECIES	SPECIMENS SCANNED
BLECHNACEAE	*Blechnopsis*	*Blechnopsis orientalis*	561
GLEICHENIACEAE	*Dicranopteris*	*Dicranopteris cadetii*	7
GLEICHENIACEAE	*Dicranopteris*	*Dicranopteris caudata*	2
GLEICHENIACEAE	*Dicranopteris*	*Dicranopteris dichotoma*	7
GLEICHENIACEAE	*Dicranopteris*	*Dicranopteris dolosa*	1
GLEICHENIACEAE	*Dicranopteris*	*Dicranopteris elongata*	1
GLEICHENIACEAE	*Dicranopteris*	*Dicranopteris emarginata*	3
GLEICHENIACEAE	*Dicranopteris*	*Dicranopteris flexuosa*	128
GLEICHENIACEAE	*Dicranopteris*	*Dicranopteris furcata*	1
GLEICHENIACEAE	*Dicranopteris*	*Dicranopteris glauca*	2
GLEICHENIACEAE	*Dicranopteris*	*Dicranopteris linearis*	834
GLEICHENIACEAE	*Dicranopteris*	*Dicranopteris nervosa*	35
GLEICHENIACEAE	*Dicranopteris*	*Dicranopteris pedata*	2
GLEICHENIACEAE	*Dicranopteris*	*Dicranopteris porosa*	1
GLEICHENIACEAE	*Dicranopteris*	*Dicranopteris pubigera*	1
GLEICHENIACEAE	*Dicranopteris*	*Dicranopteris seminuda*	6
GLEICHENIACEAE	*Dicranopteris*	*Dicranopteris splendida*	6
GLEICHENIACEAE	*Dicranopteris*	*Dicranopteris subpectinata*	7
GLEICHENIACEAE	*Dicranopteris*	*Dicranopteris taiwanensis*	3
GLEICHENIACEAE	*Dicranopteris*	*Dicranopteris tetraphylla*	6
GLEICHENIACEAE	*Dicranopteris*	*Dicranopteris underwoodiana*	1
GLEICHENIACEAE	*Gleichenella*	*Gleichenella pectinata*	247
GLEICHENIACEAE	*Sticherus*	*Sticherus bifidus*	9
JUGLANDACEAE	*Annamocarya*	*Annamocarya sinensis*	2
JUGLANDACEAE	*Carya*	*Carya alba*	53

JUGLANDACEAE	*Carya*	*Carya amara*	31
JUGLANDACEAE	*Carya*	*Carya aquatica*	22
JUGLANDACEAE	*Carya*	*Carya arkansana*	8
JUGLANDACEAE	*Carya*	*Carya buckleyi*	4
JUGLANDACEAE	*Carya*	*Carya carolinae-septentrionalis*	1
JUGLANDACEAE	*Carya*	*Carya cinerea*	1
JUGLANDACEAE	*Carya*	*Carya cordiformis*	15
JUGLANDACEAE	*Carya*	*Carya floridana*	2
JUGLANDACEAE	*Carya*	*Carya glabra*	13
JUGLANDACEAE	*Carya*	*Carya illinoinensis*	8
JUGLANDACEAE	*Carya*	*Carya laciniosa*	4
JUGLANDACEAE	*Carya*	*Carya leiodermis*	2
JUGLANDACEAE	*Carya*	*Carya mexicana*	1
JUGLANDACEAE	*Carya*	*Carya microcarpa*	6
JUGLANDACEAE	*Carya*	*Carya minima*	1
JUGLANDACEAE	*Carya*	*Carya myristiciformis*	7
JUGLANDACEAE	*Carya*	*Carya olivaeformis*	29
JUGLANDACEAE	*Carya*	*Carya ovalis*	6
JUGLANDACEAE	*Carya*	*Carya ovata*	21
JUGLANDACEAE	*Carya*	*Carya pallida*	4
JUGLANDACEAE	*Carya*	*Carya palmeri*	2
JUGLANDACEAE	*Carya*	*Carya pecan*	7
JUGLANDACEAE	*Carya*	*Carya poilanei*	5
JUGLANDACEAE	*Carya*	*Carya porcina*	47
JUGLANDACEAE	*Carya*	*Carya sinensis*	6
JUGLANDACEAE	*Carya*	*Carya sulcata*	20
JUGLANDACEAE	*Carya*	*Carya texana*	3

JUGLANDACEAE	*Carya*	*Carya tomentosa*	38
JUGLANDACEAE	*Carya*	*Carya tonkinensis*	1
JUGLANDACEAE	*Carya*	*Carya villosa*	4
JUGLANDACEAE	*Engelhardtia*	*Engelhardtia apoensis*	1
JUGLANDACEAE	*Engelhardtia*	*Engelhardtia zambalensis*	1
JUGLANDACEAE	*Engelhardtia*	*Engelhardtia aceriflora*	3
JUGLANDACEAE	*Engelhardtia*	*Engelhardtia fenzlii*	1
JUGLANDACEAE	*Engelhardtia*	*Engelhardtia mexicana*	1
JUGLANDACEAE	*Engelhardtia*	*Engelhardtia oreomunnea*	7
JUGLANDACEAE	*Engelhardtia*	*Engelhardtia parvifolia*	3
JUGLANDACEAE	*Engelhardtia*	*Engelhardtia rigida*	15
JUGLANDACEAE	*Engelhardtia*	*Engelhardtia roxburghiana*	165
JUGLANDACEAE	*Engelhardtia*	*Engelhardtia serrata*	45
JUGLANDACEAE	*Engelhardtia*	*Engelhardtia spicata*	153
JUGLANDACEAE	*Engelhardtia*	*Engelhardtia subsimplicifolia*	1
JUGLANDACEAE	*Juglans*	*Juglans ailantifolia*	11
JUGLANDACEAE	*Juglans*	*Juglans alba*	1
JUGLANDACEAE	*Juglans*	*Juglans amara*	1
JUGLANDACEAE	*Juglans*	*Juglans australis*	5
JUGLANDACEAE	*Juglans*	*Juglans boliviana*	2
JUGLANDACEAE	*Juglans*	*Juglans californica*	10
JUGLANDACEAE	*Juglans*	*Juglans cathartica*	1
JUGLANDACEAE	*Juglans*	*Juglans cathayensis*	10
JUGLANDACEAE	*Juglans*	*Juglans cinerea*	47
JUGLANDACEAE	*Juglans*	*Juglans duclouxiana*	2
JUGLANDACEAE	*Juglans*	*Juglans formosana*	1
JUGLANDACEAE	*Juglans*	*Juglans guatemalensis*	1

JUGLANDACEAE	*Juglans*	*Juglans hindsii*	3
JUGLANDACEAE	*Juglans*	*Juglans insularis*	6
JUGLANDACEAE	*Juglans*	*Juglans jamaicensis*	4
JUGLANDACEAE	*Juglans*	*Juglans major*	5
JUGLANDACEAE	*Juglans*	*Juglans mandshurica*	16
JUGLANDACEAE	*Juglans*	*Juglans meliaefolia*	2
JUGLANDACEAE	*Juglans*	*Juglans mexicana*	4
JUGLANDACEAE	*Juglans*	*Juglans microcarpa*	1
JUGLANDACEAE	*Juglans*	*Juglans mollis*	11
JUGLANDACEAE	*Juglans*	*Juglans neomexicana*	1
JUGLANDACEAE	*Juglans*	*Juglans neotropica*	4
JUGLANDACEAE	*Juglans*	*Juglans nigra*	40
JUGLANDACEAE	*Juglans*	*Juglans olanchana*	1
JUGLANDACEAE	*Juglans*	*Juglans oliviformis*	1
JUGLANDACEAE	*Juglans*	*Juglans porcina*	2
JUGLANDACEAE	*Juglans*	*Juglans pyriformis*	9
JUGLANDACEAE	*Juglans*	*Juglans regia*	101
JUGLANDACEAE	*Juglans*	*Juglans rupestris*	30
JUGLANDACEAE	*Juglans*	*Juglans sigillata*	8
JUGLANDACEAE	*Juglans*	*Juglans sinensis*	3
JUGLANDACEAE	*Juglans*	*Juglans squamosa*	1
JUGLANDACEAE	*Juglans*	*Juglans tomentosa*	3
JUGLANDACEAE	*Juglans*	*Juglans venezuelensis*	1
JUGLANDACEAE	*Platycarya*	*Platycarya strobilacea*	102
JUGLANDACEAE	*Pterocarya*	*Pterocarya caucasica*	11
JUGLANDACEAE	*Pterocarya*	*Pterocarya fraxinifolia*	35
JUGLANDACEAE	*Pterocarya*	*Pterocarya hupehensis*	2

JUGLANDACEAE	*Pterocarya*	*Pterocarya macroptera*	12
JUGLANDACEAE	*Pterocarya*	*Pterocarya paliurus*	14
JUGLANDACEAE	*Pterocarya*	*Pterocarya pterocarpa*	1
JUGLANDACEAE	*Pterocarya*	*Pterocarya rehderiana*	4
JUGLANDACEAE	*Pterocarya*	*Pterocarya rhoifolia*	14
JUGLANDACEAE	*Pterocarya*	*Pterocarya stenoptera*	86
JUGLANDACEAE	*Pterocarya*	*Pterocarya strobilacea*	1
JUGLANDACEAE	*Rhoiptelea*	*Rhoiptelea chiliantha*	16
PHYTOLACCACEAE	*Phytolacca*	*Phytolacca abyssinica*	99
PHYTOLACCACEAE	*Phytolacca*	*Phytolacca acinosa*	23
PHYTOLACCACEAE	*Phytolacca*	*Phytolacca americana*	89
PHYTOLACCACEAE	*Phytolacca*	*Phytolacca australis*	31
PHYTOLACCACEAE	*Phytolacca*	*Phytolacca bogotensis*	13
PHYTOLACCACEAE	*Phytolacca*	*Phytolacca brachystachys*	18
PHYTOLACCACEAE	*Phytolacca*	*Phytolacca chilensis*	1
PHYTOLACCACEAE	*Phytolacca*	*Phytolacca decandra*	224
PHYTOLACCACEAE	*Phytolacca*	*Phytolacca dioica*	72
PHYTOLACCACEAE	*Phytolacca*	*Phytolacca dodecandra*	137
PHYTOLACCACEAE	*Phytolacca*	*Phytolacca esculenta*	24
PHYTOLACCACEAE	*Phytolacca*	*Phytolacca goudotii*	6
PHYTOLACCACEAE	*Phytolacca*	*Phytolacca heptandra*	17
PHYTOLACCACEAE	*Phytolacca*	*Phytolacca heterotepala*	2
PHYTOLACCACEAE	*Phytolacca*	*Phytolacca icosandra*	74
PHYTOLACCACEAE	*Phytolacca*	*Phytolacca kaempferi*	1
PHYTOLACCACEAE	*Phytolacca*	*Phytolacca longespica*	2
PHYTOLACCACEAE	*Phytolacca*	*Phytolacca longifolia*	1
PHYTOLACCACEAE	*Phytolacca*	*Phytolacca octandra*	94

PHYTOLACCACEAE	*Phytolacca*	*Phytolacca polyandra*	1
PHYTOLACCACEAE	*Phytolacca*	*Phytolacca pruinosa*	27
PHYTOLACCACEAE	*Phytolacca*	*Phytolacca rivinoides*	123
PHYTOLACCACEAE	*Phytolacca*	*Phytolacca rugosa*	4
PHYTOLACCACEAE	*Phytolacca*	*Phytolacca sandwicensis*	12
PHYTOLACCACEAE	*Phytolacca*	*Phytolacca sanguinea*	2
PHYTOLACCACEAE	*Phytolacca*	*Phytolacca stricta*	1
PHYTOLACCACEAE	*Phytolacca*	*Phytolacca thyrsiflora*	38
PHYTOLACCACEAE	*Phytolacca*	*Phytolacca weberbaueri*	1
		Total	4425
