# Supplementary material for: Prospecting for rare earth element (hyper)accumulators in the Paris Herbarium using X-ray fluorescence spectroscopy reveals new distributional and taxon discoveries
Source: Ann Bot. 2024 Feb 3;133(4):573–84. doi: 10.1093/aob/mcae011 (PMC11037481; doi:10.1093/aob/mcae011)
Supplement: mcae011_suppl_Supplementary_Table_S2 [file mcae011_suppl_supplementary_table_s2.pdf]

SUPPLEMENTARY INFORMATION

Prospecting for rare earth element (hyper)accumulators in the Paris Herbarium using X-ray fluorescence spectroscopy reveals new distributional and taxon discoveries

*Léo Goudard[1], Damien Blaudez[2], Catherine Sirguey[1], Imam Purwadi[3], Vanessa Invernon[4], Germinal Rouhan[4], Antony van der Ent[1,3,5]**

[1]Université de Lorraine, INRAE, LSE, F-54000, Nancy, France

[2]Université de Lorraine, CNRS, LIEC, F-54000, Nancy, France

[3]Centre for Mined Land Rehabilitation, Sustainable Minerals Institute, The University of Queensland, Brisbane, Australia

[4]Institut de Systématique, Evolution, Biodiversité (ISYEB), Muséum national d'Histoire naturelle, CNRS, Sorbonne Université, École Pratique des Hautes Études, Université des Antilles, Paris, France

[5]Laboratory of Genetics, Wageningen University and Research, Wageningen, The Netherlands

* For correspondence. E-mail antony.vanderent@wur.nl

Supplementary Table 2. Concentrations of the different REEs in the samples analysed by ICP-AES. The concentrations are given in µg g^{-1} of dry matter. LQ corresponds to the limit of quantification of the ICP-AES instrument.

Family	Species	Specimen ID (barcode of the Paris herbarium sheet)	Sc	Y	La	Ce	Pr	Nd	Sm	Eu	Gd	Dy	Ho	Tm	Yb	Lu	LREEs	HREEs	Sum REEs
Blechnaceae	*B. orientalis*	P01576169	<LQ	661	391	477	121	612	157	53	235	160	18	5	39	5	2472	462	2934
		P01618486	<LQ	534	469	230	74	351	61	22	118	144	8	2	16	2	1741	290	2031
		P01571495	<LQ	657	1161	593	217	863	157	32	187	340	16	6	44	5	3680	598	4278
		P01619249	<LQ	1140	87	468	61	426	102	44	248	111	32	11	77	10	2328	489	2817
Gleicheniaceae	*D. flexuosa*	P01315784	<LQ	638	521	442	111	524	110	39	170	175	15	4	28	3	2385	395	2780
		P01315862	<LQ	132	62	140	14	143	44	15	63	31	<LQ	1	8	1	550	104	654
	D. linearis	P01315958	1	1130	1156	1037	311	1470	322	88	402	388	27	9	73	9	5515	908	6423
		P01474002	<LQ	699	198	629	99	657	163	54	271	119	14	3	26	3	2499	436	2935
		P00139656	<LQ	218	<LQ	5	<LQ	3	<LQ	1	10	9	2	2	8	1	227	32	259
	G. pectinata	P01316111	<LQ	564	99	210	43	203	44	32	123	76	16	4	30	3	1195	252	1447
